# Integrating participatory community mobilization processes to improve dengue prevention: an eco-bio-social scaling up of local success in Machala, Ecuador

**DOI:** 10.1093/trstmh/tru209

**Published:** 2015-01-19

**Authors:** Kendra Mitchell-Foster, Efraín Beltrán Ayala, Jaime Breilh, Jerry Spiegel, Ana Arichabala Wilches, Tania Ordóñez Leon, Jefferson Adrian Delgado

**Affiliations:** a Global Health Research Program, School of Population and Public Health, University of British Columbia, Vancouver, Canada, V6 T 1Z3; b Interdisciplinary Studies Graduate Program, University of British Columbia, Vancouver, Canada, V6 T 1Z4; c Facultad de Ciencias Médicas, Universidad Técnica de Machala, Machala, Ecuador, Km.5 1/2 Via Machala Pasaje; d Servicio Nacional de Control y Prevención de Enfermedades Transmitidas por Vectores Artrópodos, Ministerio de Salud Pública, Machala, Ecuador; e Área de Salud, Universidad Andina Simón Bolívar sede Ecuador, Quito, Ecuador, EC1701; f Área de Salud 1, Ministerio de Salud Pública, Machala, Ecuador

**Keywords:** Dengue prevention, Eco-bio-social, Ecosystem, Ecuador, Pupa per person index, Social mobilization

## Abstract

**Background:**

This project investigates the effectiveness and feasibility of scaling-up an eco-bio-social approach for implementing an integrated community-based approach for dengue prevention in comparison with existing insecticide-based and emerging biolarvicide-based programs in an endemic setting in Machala, Ecuador.

**Methods:**

An integrated intervention strategy (IIS) for dengue prevention (an elementary school-based dengue education program, and clean patio and safe container program) was implemented in 10 intervention clusters from November 2012 to November 2013 using a randomized controlled cluster trial design (20 clusters: 10 intervention, 10 control; 100 households per cluster with 1986 total households). Current existing dengue prevention programs served as the control treatment in comparison clusters. Pupa per person index (PPI) is used as the main outcome measure. Particular attention was paid to social mobilization and empowerment with IIS.

**Results:**

Overall, IIS was successful in reducing PPI levels in intervention communities versus control clusters, with intervention clusters in the six paired clusters that followed the study design experiencing a greater reduction of PPI compared to controls (2.2 OR, 95% CI: 1.2 to 4.7). Analysis of individual cases demonstrates that consideration for contexualizing programs and strategies to local neighborhoods can be very effective in reducing PPI for dengue transmission risk reduction.

**Conclusions:**

In the rapidly evolving political climate for dengue control in Ecuador, integration of successful social mobilization and empowerment strategies with existing and emerging biolarvicide-based government dengue prevention and control programs is promising in reducing PPI and dengue transmission risk in southern coastal communities like Machala. However, more profound analysis of social determination of health is called for to assess sustainability prospects.

## Introduction

Dengue is a major public health problem in Ecuador,^1,2^ with a diverse array of contributing factors and a lack of intersectoral organization within governance systems exacerbating it. Ecuador has identified all four dengue serotypes within its borders and the vector, *Aedes aegypti*, is distributed throughout the tropical, subtropical regions of the country as well as all of its islands.^1,3,4^ Moreover, there has been poor integration of approaches to dengue prevention; especially with regard to primary education, social mobilization, poverty and deficiency of basic public services as determinants for dengue transmission.^2,5^ Dengue control programs in Ecuador, as with many countries in the Americas, rely on government application of temephos larvicide, deltamethrine adulticide and *Bacillus thuringiensis israelensis (bti)*-based biolarvicide.^6–8^ Present vector control strategies have not succeeded in maintaining vector populations below the levels required to eliminate dengue transmission in endemic regions; however, emerging collaborative work in Machala has developed new integrated approaches to dengue management that focus on community empowerment and determinants of health as well as vector source reduction.

It has been consistently documented that the incidence of dengue fever/severe dengue has increased at an alarming rate in the Latin America and Caribbean region, with epidemics becoming larger and more frequent.^2,5,6,9–12^ The relation of this trend to current ‘global change’ factors (e.g., climate change, urbanization, poverty) has drawn further attention to the need to address this growing health threat. With 50–100 million worldwide cases of dengue fever occurring annually and an estimated 2.5 billion people living in areas endemic for dengue virus transmission,^13–15^ the development of effective control and prevention strategies based on the best science and context-specific knowledge is urgently required. Both ecological and social system challenges must be addressed; it is essential that ‘bridges’ to effective control and prevention be built by facilitating participatory, community-based and transdisciplinary approaches^16^ to overcome institutional barriers.

There is a growing movement and evidence base to support the fundamental change in vector control and dengue prevention, both in practice and in policy, to a community-based participatory model centered around an eco-bio-social approach.^6–19,9^ The ecosystems approach to human health (ecohealth) has been a successful eco-bio-social model used in vector control programs at a local level.^16,20,21^ Communities in Machala have been vocal about the need for alternative vector control and dengue prevention strategies,^2,5,12^ prompting research by epidemiologists, public health officials, vector control officials and university researchers to explore and pilot new prevention efforts based on the eco-bio-social paradigm or the ecohealth, which views human health as a product of and determining force on the ecosystems that people live and work in. While Ecuador reports implementing the Integrated Management Strategy for the Prevention and Control of Dengue (IMS-Dengue; or the Spanish acronym EGI-Dengue^22^) in 2010,^4^ common challenges remain in ensuring sustainability of social mobilization and social communication elements, as well as operational challenges in assuring adequate coverage of vector source reduction campaigns and reducing dependence on insecticides.^8^ The work presented here seeks to provide applicable, locally contextualized operational research on these fronts.

This collaboration's overarching objective was to better understand the effectiveness and feasibility of applying an integrated ecohealth approach to dengue prevention and control in endemic urban and peri-urban settings marked by infrastructural weaknesses. It builds on two separate participatory action research pilot studies based in Machala^11^ that established the feasibility and efficacy of conducting community-based interventions to promote the prevention and control of dengue applying an ecosystem approach to human health. The first study^11^ applied a comprehensive dengue elementary school education program (DESE), including classroom knowledge, practical skill development and application (Arichabala-Wilches A, Universidad Técnica de Machala, unpublished data). The second study^11^ evaluated a 6-week participatory ‘Clean Patio and Safe Container’ (CPSC) strategy focusing on removing discarded or unused containers/materials from patios, and brushing out and covering ground-level tanks to prevent *Ae. aegypti* breeding sites within the peri-domestic area (Beltrán-Ayala F, Universidad Técnica de Machala, unpublished data). Both small-scale pilot studies documented improvements to house index (HI; proportion of inspected houses positive for the presence of *Ae. aegypti*, expressed as a percentage) and Breteau index (BI; number of containers positive for immature *Ae. aegypti* per 100 houses inspected) measures at the household level, as well as the interest of diverse stakeholders for active involvement in addressing the complex community health challenge of dengue. Integrating these two locally successful approaches and applying them to serve a larger population at risk may provide insights into sustainability and operational challenges experienced with IMS-Dengue in Machala. Further, this study provides the timely opportunity to explore the potential use of the pupa per person index (PPI) as a vector control outcome measure to complement the current use of HI and BI outcome measures by the Ministry of Health National Vector Borne Disease Control Service (SNEM).

Here we examine the effectiveness of applying an integrated community-based approach to prevent and control dengue in a vulnerable endemic setting and analyze its implementation in comparison with current government programs, and we investigate the effectiveness and feasibility of scaling up an ecosystem approach to dengue prevention and control amid the resurgence of dengue in southern Ecuador.

## Materials and methods

### Study site

Machala is the capital of El Oro (population ∼300 000; 72% urban population), the southernmost province on Ecuador's Pacific coast where 41.4% of the population lives at or below the poverty line. Machala's many peri-urban and semi-rural neighborhoods are characterized by unplanned urbanization, basic infrastructure and provision of basic services, and low housing quality. *Ae. aegypti* are present year-round with high vector indices (HI:12.6%, BI: 18.3). Essentially, Machala exists in a perpetual state of risk for outbreaks and epidemic transmission of dengue with frequent outbreaks and an average incidence of 28.5 cases per 10 000 popluation.^5^ *Aedes* indices historically peak between February and April, following the precipitation trend of the rainy season (October to March) and reported cases of dengue routinely closely follow this trend. Recurrent outbreaks and epidemics are becoming more frequent in Machala due to local climatic variation manifesting in lengthened and less predictable rainy seasons.^5,12^

### Sampling design

We used a two-stage sampling design (documented in a previous publication^6^) to select and include 20 clusters of 100 households in a randomized controlled cluster trial (RCCT). Using a satellite image map of Machala generated by Google Earth^23^, we generated a base map of city blocks, residential areas, roadways, public buildings and green spaces within municipal jurisdictional boundaries upon which to build the study design. Using the PPI as the outcome measure,^6^ and assuming a PPI value of 0.4 and an expected reduction of 75% after intervention, the power of estimation at a 5% level with this sample size is larger than 90%, comfortably covering possible non-responses.

Comparative analyses for the RCCT were based on data collected through the use of both entomological and household surveys informed by the Theory of Planned Behaviour.^24^ Gathered information describes specific vector densities within selected areas, to measure vector densities using PPI, HI and BI, and the stated knowledge attitudes and practices of residents in the cluster areas with regards to water management and environmental risk factors for dengue transmission. Pre-intervention baseline surveys were done in March 2012, and post-intervention surveys done in November 2013; both are rainy season measures. These full methodologies are described in an earlier publications.^6^ The paired design matched clusters sharing similar overall (average) socio-economic indicators and baseline PPI, with random selection of the intervention site.

### Integrated intervention strategy

Within the overall objective of comparatively analyzing effectiveness and sustainability of an eco-bio-social ecohealth-style approach to dengue prevention and control with conventional government-mandated programs, 10 clusters were randomly assigned for the intervention and 10 for the control. The integrated intervention strategy (IIS) was introduced in each intervention site to promote acculturation to the eco-bio-social approach and included a DESE (Beltrán-Ayala F, Universidad Técnica de Machala, unpublished data) coupled with a CPSC (Arichabala-Wilches A, Universidad Técnica de Machala, unpublished data) program incorporating social mobilization strategies:

#### DESE

From October 2012 to November 2013, DESE was rolled out in a step-wise way in schools and community centers for children aged 8–12 years in participant households. Four 30-minute classes over 2 weeks taught modules about the symptoms of dengue, the dengue transmission cycle, the lifecycle of the mosquito *Ae. aegypti* and laboratory observation of the different life stages of the vector. This was followed by a practical learning session in a neighborhood location providing practical education enabling children to practically apply their knowledge in their own context. Classroom sessions were repeated every 6 months, with once-monthly follow-up practical pupal collection ‘homework’ assignments where children collected mosquito pupae specimens from their own homes and submitted to DESE personnel for identification at the SNEM vector entomology lab. Classroom and follow-up assignments were administered by health educators, public health practitioners and frontline vector control personnel, and results were returned to the community within 10 days. The expected outcome of the practical sessions is that children are able to identify, record and control (i.e., empty water or request control measures from a parent) mosquito-breeding sites in their immediate environment. This program is designed with specific elements for child empowerment and social mobilization for children as emerging health leaders in their own communities.

#### CPSC

Using community empowerment and social mobilization to eliminate *Ae. aegypti* breeding sites in peri-domestic areas, community volunteer CPSC activators (one activator per each 10–15 participant houses, 4 h per month) were recruited from within each cluster. SNEM health promoters supported CPSC activators by coordinating house inspection dates and collecting datasheets for project databases; SNEM employees did not accompany activators on their house visits once training was complete. Once-monthly community-directed visits to participant homes cataloged the state of the patio based on three criteria: (1) cleanliness (absence of discarded containers, solid waste, animal feces and plastics), (2) weed and plant removal (elimination of mosquito sanctuary in wild plants) and (3) orderliness (stored containers are protected from accumulating water, stored water is changed regularly, controlled with larvicides and in well-maintained containers). CPSC data collection recorded condition of the patio, identified and enumerated breeding sites positive for water, larva and/or pupae, and numbers of pupae present in each participant home; monthly visit forms were processed by SNEM health promoters. Households repeatedly showing poor indices over a 3-month period were flagged by community inspectors for support by vector control personnel to collaboratively seek solutions to address underlying dynamics of identified dengue risks. Community CPSC activators were recruited in October 2012, with program rollout unfolding in eight of the ten intervention clusters from an initial fully supported month-long program launch and scaled-up ‘pilot’ in November 2012. This initial intensive capacity-building and human resource investment ensured communities were trained, willing and able to run CPSC efforts without continued intensive support from vector control and Ministry of Health staff; it was also used to assess levels of community capacity in this respect. The majority of communities independently carried forward the CPSC program using built capacity. Continued capacity building and human resource investments for the duration of the implementation window were offered to communities with higher risk factors and special considerations from November 2012 to November 2013.

#### Control clusters with government-mandated strategy

Conventional SNEM dengue prevention and control strategies included the use of temephos larvicide (Abate) as a preventive breeding-source control measure, coupled with routine (blanket mosquito control) and reactive (targeted *Ae. aegypti* control in response to suspected and confirmed dengue cases) intradomiciliary deltamethrine fogging using backpack sprayers, and neighborhood vehicle-mounted malathion fogging. Once or twice yearly household visits by vector control workers are conducted to monitor entomological indices. However, from April 2013 SNEM dengue control strategies were significantly altered in Machala by the rollout of a new federally mandated *bti-*biolarvicide-based control program characterized by a huge increase in government vector control worker presence, educational and technical intervention in individual homes and communities. In this revised monitoring and control strategy, a vector control technician/frontline worker visits each home in Machala twice per month to educate household members on mosquito-breeding source reduction, to apply biolarvicide, educate around biolarvicide application, leave a small quantity of biolarvicide for domestic use until the next visit, and inspect/record/educate/eliminate mosquito-breeding source containers. Although the new strategy does incorporate eco-bio-social elements, it is currently in the pilot stage and full integration of these elements has not been achieved, nor has full coverage of Machala to date. The biolarvicide-based program was incrementally rolled-out and implemented during the intervention period resulting in a temporally and geographically varied mix of baseline control programs in control clusters. This called for a more sophisticated analysis than had been originally planned, to ensure that the intervention's effect would focus on those cluster pairings that remained faithful to the study design.

### Statistical analysis

Using SPSS statistical software version 21.0, we modelled the pre- and post-intervention pupal counts by using a Poisson regression model, accounting for cluster pairing. The Poisson regression model links the logarithm of the count as a linear function of independent variables. Using intervention/control status (a binary indicator for community type where intervention=1 for the intervention clusters and=0 for the control clusters) as the independent variable, our model is:

$$\text{Log}(\text{pupa rate})=\text{intercept}+{\text{b}}^{*}\;\text{intervention}$$

The effect of the intervention compared to the control is expressed as the difference in the log (pupa rate) between the two models where ‘b’ is the additive difference on a log scale.

We then applied a generalized estimating equations (GEE) analysis that is appropriate for a randomized cluster design study.^25,26^ To compare observed versus expected pupal distributions within each cluster pairing, we also applied a χ^2^ test. Due to the complexities of IIS implementation introduced by the concurrent rollout of new Ministry of Health biolarvicide mosquito control programs, we organized our analysis to highlight those clusters where the original study design was successfully applied and where there were no confounding municipal interventions, while considering the effects of the biolarviciding program as a confounder in those sites where it had been introduced. This effectively excluded clusters where not all the interventions had been introduced as planned as a result of implementation personnel being overextended with the biolarviciding program (i.e., pairs 1, 2 and 4), and pair 7 (due to cluster 2, Simón Bolívar) where effects were observed due to substantially improved infrastructure and basic services being introduced during the study period.

### Ethical considerations and partnerships

Informed consent was obtained for each household and child participant recruited to the study prior to their participation. This collaborative work partnered communities, researchers and governmental decision-makers to validate and approve the integrated intervention strategy: Ministry of Education, Ministry of Health, Ministry of Health National Vector Borne Disease Control Service (SNEM), Provincial Government of El Oro, Municipal Government of Machala, Parish Councils and Community Councils.

## Results

A total of 1986 households participated within the 20 cluster RCCT, with 4014 residents in intervention households and 3886 in control households (Table 1). Overall, while control and intervention communities did not have statistically significant differences in their PPI values prior to the intervention, significantly reduced overall PPI values were observed in the intervention clusters following the IIS implementation period (Table 1).

**Table 1.** Overall study results by treatment using the pupa per person index (PPI)

			PPI (2012)	PPI (2013)	% change 2012–2013	P value	Persons	Pupa (2012)	Pupa (2013)
Pair no.	Cluster no.	Neighborhood							
High socioeconomic									
1	4 (I)	9 de Octubre	0.159	0.027	–83.0%	<0.001	477	76	13
	10 (C)	Central	0.157	0.195	24.2%		364	57	71
2	6 (I)	18 de Octubre	0.870	0.037	–95.7%	<0.001	376	327	14
	15 (C)	Velazco Ibarra	0.185	0.193	4.3%		357	66	69
3	13 (I)	3 de Noviembre	0.477	0.038	–92.0%	<0.001	394	188	15
	7 (C)	Asoc. Empleados Municipales	0.157	0.427	172.0%		415	65	177
Medium socioeconomic									
4	5 (I)	24 de Mayo	0.993	0.176	–82.3%	0.259	455	452	80
	16 (C)	El Bosque Sector 4	0.359	0.082	–77.2%		415	149	34
									
5	19 (I)	Mario Minuche	0.252	0.052	–79.4%	<0.001	421	106	22
	1 (C)	24 de Julio	0.952	0.738	–22.5%		378	360	279
									
6	8 (I)	Venezuela	0.702	0.003	–99.6%	<0.001	372	261	1
	11 (C)	Manuel Encalada	0	0.206	inf +		330	0	68
Low socioeconomic									
7	9 (I)	Luz de América	0.008	0.137	1612.5%	Negative	393	3	54
	2 (C)	Simón Bolívar	1.123	0.123	–89.0%	<0.001	389	437	48
8	3 (I)	Martha Bucaram	0.739	0.210	–71.6%	0.603	395	292	83
	18 (C)	7 de Marzo	1.829	0.561	–69.3%		403	737	226
9	12 (I)	25 de Junio	0.471	0.080	–83.0%	<0.001	350	165	28
	20 (C)	El Retiro	0.553	0.269	–51.4%		398	220	107
10	14 (I)	Sauces #1	0.609	0.010	–98.4%	<0.001	381	232	4
	17 (C)	24 de Septiembre	2.483	1.362	–45.1%		437	1085	595
Totals (all 10)									
Machala totals			0.668	0.252	–62.3	<0.001	7900	5278	1988
Intervention (I)			0.524	0.080	–85.1%	**	4014	2102	314
Control (C)			0.817	0.353	–47.2%		3886	3176	1674

**: statistically significant; inf+: SPSS output for an incalculable positive % increase for the PPI readings from 2012 to 2013. As there were no pupae collected from this neighborhood in 2012, and a PPI of 0.206 in 2013, the program treats this as mathematically problematic in using a 0 as the starting point percentage.

p Values calculated by applying χ^2^ test comparing observed versus expected values, based on the 2012 pupa per person index distributions.

Analysis of the effects of the intervention in all the clusters that had been enrolled in the study pointed to a potential effect of the intervention that was of equivalent impact as the annual variation that had been observed in the study period, which was just outside of statistical significance (Table 2; p=0.121). However, when only the cluster pairings that had remained consistent with the study design were included, the effect of the intervention was stronger (2.2 OR, 95% CI: 1.2 to 4.7) compared to comparison clusters and statistically significant (p=0.015), with no statistical significance associated with the effects of the biolarviciding program in the study areas (Table 2).

**Table 2.** Effects on the pupa per person index (PPI) pre- and post-intervention

Effect	Odds ratio	95% CI	P value	B	SE	95% CI
All cluster pairs						
Annual change	1.6	1.0 to 2.6	0.033	0.491	0.2305	1.041 to 2.568
Intervention	1.7	0.9 to 3.3	0.121	0.528	0.3401	–0.054 to 1.302
Biolarviciding	0.4	0.1 to 1.2	0.091	–0.929	0.5499	0.135 to 1.160
Cluster pairs where study design applied^a^						
Annual change	1.8	1.0 to 2.8	0.011	0.595	0.2319	0.139 to 1.051
Intervention	2.2	1.2 to 4.7	0.015	0.801	0.3535	0.155 to 1.447
Biolarviciding	0.5	0.1 to 1.7	0.286	–0.684	0.6066	–2.358

Estimated with a generalized estimating equation models with binomial link function.

^a^ Excluding clusters # 1,2,4 where all interventions were not carried out; and #7 where major infrastructure improvements took place.

A total of 230 children in the 10 intervention clusters participated in the DESE arm of the IIS (Table 3), with a reduction in both HI and BI in their own households. A total of 729 households participated in the CPSC program in eight intervention clusters (Table 4); with five of these clusters carrying forward the CPSC independently throughout the implementation window and three of these clusters using capacity-building support and human resources investments from vector control staff and Ministry of Health frontline workers. Overall PPI measures were used as outcome measures for CPSC.

**Table 3.** Number of child participants and percentage of total eligible neighborhood children who participated in the dengue elementary school education program by neighborhood

Cluster	Neighborhood	No. child participants (%)	Pre-intervention (2012)		Post-intervention (2013)	
			House index (%)	Breteau index	House index (%)	Breteau index
3	Martha Bucaram	33 (62.2)	3.0	3.0	0.0	0.0
5	24 de Mayo	32 (71.1)	3.1	12.5	0.0	0.0
6	18 de Octubre	6 (18.2)	16.7	16.7	0.0	0.0
8	Venezuela	14 (48.2)	0.0	0.0	0.0	0.0
9	Luz de Ámerica	34 (77.2)	8.8	8.8	0.0	0.0
12	25 de junio	11 (47.8)	36.4	45.5	0.0	0.0
13	3 de Noviembre	19 (70.3)	5.3	10.5	5.3	5.3
14	Sauces #1	23 (79.3)	8.7	21.7	0.0	0.0
19	Mario Minuche	19 (73.1)	5.3	5.3	0.0	0.0
Total		230 (66.1)	13.0	29.6	1.3	1.7

**Table 4.** Intervention clusters with implemented clean patio and safe container social mobilization and source reduction program

Cluster	Neighborhood	November pilot+independent community-driven follow-up	November pilot+project supported follow-up	Participant households
3	Martha Bucaram	✓		95
8	Venezuela	✓		81
9	Luz de América	✓		91
12	25 de Junio	✓		100
13	3 de Noviembre	✓		94
14	Sauces #1		✓	86
19	Mario Minuche		✓	82
Total		5	3	729

## Discussion

The eco-bio-social approach is particularly promising for dengue prevention and control as the presence of the vector and its density depends on the human population, human behavior and how these affect the surrounding environment.^27^ Much of the evidence in support of ecohealth and community-based interventions describes their implementation efficacy and acceptability, but does not address their effectiveness and sustainability.^27,28^ In many cases, ecohealth interventions are short-lived, localized to particular communities, are undertaken without provisions for scalability or involvement in the policy-making process and the indices used to measure the impact of the projects do not accurately reflect dengue transmission risk.^27,29^ The cluster randomized trial has been adopted as a methodology that strengthens the results of ecohealth interventions and combined with the use of PPI for paired clusters (in lieu of HI or BI) increases generalizability and improves the evaluation of efficacy of the programs through a more accurate estimation of adult vector abundance.^27,30^ Our multifactorial approach, integrating social mobilization, addressing proximal environmental determinants of dengue transmission risk (peri-domestic vector breeding site reduction) and engagement in intersectoral collaboration, has shown a significant reduction in PPI at the scaled-up level (Beltrán-Ayala F and Arichabala-Wilches A, unpublished data).

Each cluster had its own unique context and dynamics that affected IIS implementation resulting in varying degrees of eco-bio-social intervention success. Community-driven implementation of the CPSC in two cluster communities required continuing capacity-building support and human resource investment: Sauces #1 (cluster 14), a neighborhood frequently flooding, poor infrastructure and solid waste collection, and significant public insecurity; and Mario Minuche (cluster 19), a peri-urban neighborhood characterized by inadequate housing, poor solid waste collection bordering on an uncovered canal. Implementation of IIS following original experimental design was not achieved in pairs 1, 2 or 4 due to saturation of skilled human resources for vector control and the incremental rollout of the SNEM *bti*-biolarvicide dengue prevention program in the study area and adjacent areas.

Further, the significant reduction of PPI in control cluster 2 (Simón Bolívar; pair 7) is a direct result of long-standing and ongoing community-based political action by the neighborhood council and neighborhood president that saw the realization of a full neighborhood revitalization project in collaboration with the Municipal Government of Machala during the intervention period. Prior to revitalization, Simon Bolívar's dengue transmission risk indicators were high (PPI 1.123), with many residents lacking potable water in their homes, significant gang activity and social insecurity in the neighborhood, poor and sometimes lacking roadway infrastructure and garbage collection, and inadequate housing. The revitalization included basic infrastructure such as installation and improvement of sewers and storm sewers, roads, sidewalks, lighting in public spaces, a children's playground, increased police presence, improved garbage collection, and improvements to private residences and properties. As a result, and without the implementation of the IIS in their neighborhood, PPI was reduced to very low or minimal risk. This empowered intersectoral collaboration between the community of Simón Bolívar and the Municipal Government of Machala vividly illustrates the crucial role that provision of basic services, basic infrastructure, and considerations for human and community security, play in establishing healthy communities and reducing the incidence of diseases of poverty like dengue fever and severe dengue. The increase in PPI for cluster 9 is a result of localized flooding during November 2013 in the neighborhood.

IIS implementation was very successful in cluster 14 (Sauces #1) with high community enthusiasm and sustained involvement. This, along with the effects of localized flooding in 2012 and a very dry season in 2013, resulted in a significant reduction of PPI. Also interesting in this cluster pair is the dramatic reduction of PPI in control cluster 17 (24 de Septiembre). This highly organized and motivated community capitalized on project presence to leverage support for other ongoing efforts to improve well-being and human security in their neighborhood. Community leaders routinely assemble and coordinate efforts to address community issues and garner support from the Municipal Government of Machala as well as the Ministries of Health, Environment and Education; there were a number of these efforts that coincided with interests of the IIS in reducing dengue transmission risk. Two particular efforts were of interest in this respect: an improved community-driven solid waste removal campaign partnered with the Municipal Department of Environment to reduce the presence of containers (possible *Ae. aegypti* breeding sites), and an infrastructure project partnered with the Municipal Department of Public Works to fill in the lagoon in their neighborhood square to reduce standing water.

The unforeseen rollout of a new biolarvicide dengue prevention program complicated IIS implementation efforts within the community-based project model by saturating existing skilled human resources and introducing logistical complications. Increased community interface time within the biolarvicide program (two in-home visits per month by a vector control technician and/or frontline worker) affected the CPSC empowerment strategy and original scale-up recommendations in three ways: (1) shifts in Ministry of Health vector control programs and services increased governmental intervention in communities that do not depend on community-driven empowerment strategies; (2) they introduce a more ecologically sensitive model of dengue prevention with the reduction of insecticides (addressing a major community concern); and (3) offer a parallel stream for community involvement, time, enthusiasm and good-will toward dengue prevention and control. While a complication, this experience introduced a novel opportunity to consider the interface of alternative strategies.

The success of our proposed IIS dengue prevention program during biolarvicide program rollout indicates a complementarity of efforts: both programs respond to community concerns of reducing insecticide use in domestic and peri-domestic environments, increasing skilled Ministry of Health human resource investment to reduce dengue incidence in affected communities, and improving health education and dengue prevention through positive behavior change in homes, neighborhoods and schools. However, it is the IIS strategy that is observed the more effective approach for reducing PPI levels. Nevertheless, integrating community-driven empowerment strategies like DESE and CPSC within the robust structure and function of the biolarvicide program could enhance synergy between parallel streams and capitalize on emerging momentum and progress in dengue prevention and control in southern Ecuador.

### Conclusions

An integrated eco-bio-social approach to reducing PPI as a useful outcome measure of dengue transmission risk has been successful in Machala, Ecuador, although difficulties encountered in smoothly implementing this study as it was designed suggest that it would still be valuable to conduct further implementation trials as part of scaling-up activity. Additionally, our results suggest that local successes in ecosystems approaches to dengue prevention can be successful if that process is pursued in partnership with communities, incorporates an element of social mobilization and empowerment, and emphasizes intersectoral collaboration. Examples outlined in this paper of intersectoral collaboration between communities, control programs and municipal governments underscore the importance of integrating efforts, resources, momentum and empowerment from all actors involved with and concerned with dengue prevention and control in individual communities and over larger municipal and regional endemic zones.
